# Disaster Microbiology—a New Field of Study

**DOI:** 10.1128/mbio.01680-22

**Published:** 2022-08-03

**Authors:** Daniel F. Q. Smith, Arturo Casadevall

**Affiliations:** a W. Harry Feinstone Department of Molecular Microbiology and Immunology, Bloomberg School of Public Health, The Johns Hopkins University, Baltimore, Maryland, USA; Ohio State University

**Keywords:** disaster microbiology, climate change, extreme weather, microbial adaptation, natural disaster, human-made disaster, bioremediation, emerging pathogens

## Abstract

Natural and human-made disasters can cause tremendous physical damage, societal change, and suffering. In addition to their effects on people, disasters have been shown to alter the microbial population in the area affected. Alterations for microbial populations can lead to new ecological interactions, with additional potentially adverse consequences for many species, including humans. Disaster-related stressors can be powerful forces for microbial selection. Studying microbial adaptation in disaster sites can reveal new biological processes, including mechanisms by which some microbes could become pathogenic and others could become beneficial (e.g., used for bioremediation). Here we survey examples of how disasters have affected microbiology and suggest that the topic of “disaster microbiology” is itself a new field of study. Given the accelerating pace of human-caused climate change and the increasing encroachment of the natural word by human activities, it is likely that this area of research will become increasingly relevant to the broader field of microbiology. Since disaster microbiology is a broad term open to interpretation, we propose criteria for what phenomena fall under its scope. The basic premise is that there must be a disaster that causes a change in the environment, which then causes an alteration to microbes (either a physical or biological adaptation), and that this adaptation must have additional ramifications.

## Disaster Microbiology

A disaster is defined broadly by Merriam-Webster as “a sudden event, such as an accident or a natural catastrophe, that causes great damage or loss of life,” (1) and can be caused by natural hazards (geophysical, hydrological, climatological, meteorological, or biological) or human-made hazards (conflicts, industrial accidents, transportation accidents, environmental degradation, or pollution), according to the International Federation of Red Cross and Red Crescent (2). Disasters impose immense technological, physical, financial, psychological, and health burdens upon the people affected and threaten our infrastructure around the globe. The term “disaster microbiology” was used in a recent report by the American Society for Microbiology on the effects of climate change on microbes and defined as a “proposed field of study focused on the microbial impacts from severe storms and natural disasters” (3), which inspired this article. In the history of science, scientific fields emerge when communities form with common scientific interests (4). Formal establishment of a field also creates an environment in which there is “creation of cohesive communities, preservation of information, [and] establishment of normative standards” (4). At this time, the field of disaster microbiology is not yet recognized as a distinct entity, but in this article, we argue that sufficient knowledge and research exist to create a new tent that would house these efforts and promote their further development and collaboration across currently separated disciplines. The focus of this article is anthropocentric simply because we know most about human consequences and because as humans we are necessarily concerned about human well-being, but it is important to note that any disaster also affects all other species living in the area.

The emergence of the field of disaster microbiology would allow microbiologists, epidemiologists, ecologists, environmental engineers, and infectious disease and other experts to organize their research around the shared goal of mitigating microbial consequences from such events and to learn the basic science that accompanies microbial adaptation to catastrophe. This field would seek to understand how natural and artificial disasters drive adaptation of environmental microbes and change microbial interactions with humans, and how both combined have implications for human health. As public health researchers and disaster preparedness organizations make recommendations for plans to treat and avoid disaster-incited infectious disease outbreaks, disaster microbiology could inform them on how microbes respond to disaster and which are at highest risk of causing outbreaks depending on disaster type, geography, and socioeconomic conditions.

## NATURAL DISASTERS

Natural disasters have long been seen as a harbinger of public health crises, including those related to infectious disease (5). Cyclones, tsunamis, flooding, and tornadoes can each place humans into direct contact with microbes with which they would not have otherwise come into contact. In instances where the event causes wounding, there is the added risk of infected wounds and bloodstream infections. For example, tornadoes in Joplin, Missouri resulted in widely reported cases of mucormycosis as victims were traumatically inoculated with the fungus *Apophysomyces trapeziformis* after being wounded by debris (6–8).

Anthropogenic climate change is causing rapid environmental changes. Climate change increases the frequency of tropical cyclones, extreme heat, tornadoes, droughts, and wildfires. In addition to increased disaster-mediated exposure of hosts to pathogenic microbes caused by climate change, climate change could accelerate the disaster-driven evolution of microbes in response to stressors such as heat waves, droughts, chemical or toxic waste, and wildfire. Microbial stress-response factors that allow microbes to survive environmental change can also affect their interactions with the mammalian immune system. Additionally, with increasing frequency and duration of heatwaves, microbes will come under increasing selection for their ability to persist and survive at higher temperatures (9).

### Flooding disasters.

Flooding can follow from several types of natural disasters including rainstorms, tropical cyclones, and tsunamis. These all result in the inundation of normally dry land with water from rivers, streams, marshland, ocean, or rainwater runoff, which can subsequently cause damage as it enters homes, businesses, and roadways. In 2020, flooding events affected nearly 35 million people worldwide (10). Flooding increases exposure to microbes by disrupting soil and bringing soil microbes into direct contact with flooding victims. Tropical cyclones are associated with increased risk of respiratory infections, gastrointestinal infections, and other communicable diseases (11–13). Flooding and water damage to homes can promote the growth of toxin-producing molds such as those that occurred in many New Orleans homes following Hurricane Katrina, resulting in respiratory symptoms (14, 15). Filamentous fungi, including Aspergillus spp. also proliferated within water-damaged homes in Puerto Rico following Hurricane María (16). Molds growing within damp or water-damaged homes is sometimes associated with increased risk of asthma in children and adults (17). Such molds can also take residence in other buildings and is associated with “sick building syndrome,” in which individuals fall ill from several causes, including chronic exposure to fungal-derived toxins (18, 19). Tsunamis have been associated with other fungal infections, namely, Fusarium spp., *Mucor* spp., Aspergillus fumigatus, and Scedosporium apiospermum during the 2004 Indian Ocean tsunami and 2011 Japanese tsunami (20, 21). The 2011 earthquake and tsunami in Japan was associated with increased cases of legionellosis due to inhalation of droplets of soil-contaminated water that contained *Legionella* spp. bacterium and Aspergillus fumigatus (22). In addition, flooding events can lead to wastewater treatment plants being overrun, causing spillage of human waste and thus more waste-contaminated floodwater (23). While a common fear following natural disaster, decomposing bodies of victims do not typically cause the spread of disease or contamination of water supplies (24–26). Floodwaters could introduce microbes to an environment where they can establish a niche to cause disease in the decades after the disaster is over. It has been theorized that in 1964, the tsunami caused by the Great Alaskan Earthquake spread the fungus Cryptococcus gattii from its aquatic niche in the Pacific Ocean onto land in the Pacific Northwest and Vancouver Island, where it later emerged to cause outbreaks starting in 1999 (27).

In tropical regions, flooding can allow increased proliferation of disease vectors such as malaria-carrying *Anopheles* spp. mosquitoes or yellow fever-, dengue-, and Zika-causing *Aedes* spp. mosquitoes, and thus transmission of more vector-borne disease (28, 29). Mosquito-borne diseases already account for more than 700,000 deaths per year, despite extensive mitigation efforts; the added burden of natural disasters and possible increased breeding ground can increase these deaths and increase the number of people at risk globally (30).

### Dust-related disasters.

Other natural disasters relevant to disaster microbiology are those that involve the spread of dust into the air, which can often contain pathogenic microbes, such as tornadoes, earthquakes, dust storms, and droughts. Incidence of bacterial and fungal pneumonias increased in areas that had more frequent tornadoes (31) A windstorm causing soil dispersion in California triggered a widespread outbreak of the soil-dwelling fungus *Coccidioides immitus*, which resulted in over 115 cases and 8 deaths (20, 32). Similarly, there were 203 reported cases of coccidioidomycosis following the 1994 Northridge Earthquake in California, associated with dust clouds and high incidence of landslides (33). Like earthquakes and dust storms, coccidioidomycosis was associated with drought conditions, when the soil is dry and prone to forming dust (34). As global temperatures and drought frequency increase in subsequent decades, the geographic range of coccidioidomycosis is projected to expand (35). Drought often leads to water conservation and altered habits that can lead to diseases caused by organisms such as Giardia parasites and Escherichia coli (36, 37).

### Wildfires.

Wildfires can also be agents for dispersal of microbes, particularly those that are found in the soil, and proximity to wildfires is associated with increased risk of upper and lower respiratory infections (38–40). Importantly, wildfires change the bacterial and fungal composition of the soil, which, over time, selects for microbes that have stress-resistant spores and a relative heat tolerance (41). These stress-resistant adaptations can cause issues for humans, where spore walls and adaptation to heat stress facilitate microbial infection at human body temperature. The smoke from fires can aerosolize and transport viable fungi and bacteria, with a microbial concentration in the smoke far greater than the ambient air (42). The smoke from fires can transport plant pathogens and beneficial soil and plant microbes over long distances. The study of how microbes interact with fire and smoke was termed “pyroaerobiology” by the authors (42).

### Heat extremes.

Heat waves and extreme heat events are high-stress events for typical organisms. Extreme heat is associated with other natural disasters such as drought, wildfire, dust storms, and even tropical cyclones. Urban areas are particularly vulnerable to heat, due to the “heat island” effect caused by lack of green space, airflow, and building materials (43–45). For microbes, heat can drive adaptation for thermotolerance, permitting survival at human body temperature for microbes that do not already have that capability (46). The increasing thermotolerance of microbes is particularly an issue in the emergence of fungal pathogens since many environmental fungi grow best below 37°C, or human body temperature; in fact, this thermal exclusion zone may be one reason that fungal diseases are historically uncommon, especially for those without compromised immune systems (47). While the 20th century saw a burgeoning of fungal pathogens as one fungal barrier—the immune system—was weakened due to the human immunodeficiency virus (HIV) pandemic and the advent of immunosuppressive medical treatments, the 21st century may see a rise of fungal diseases as fungal species with pathogenic potential adapt to higher temperatures and breach mammalian thermal barriers (48). Global warming has already been proposed as a mechanism for how Candida auris gained temperature tolerance and jumped from a strictly environmental niche to one that can cause human disease (9).

## HUMAN-CREATED DISASTERS

While the definition of disaster microbiology used by the American Society for Microbiology report focused on natural disasters (3), human-made disasters can also alter and change microbes in a way that would fall under “disaster microbiology.” War, terrorism, mass shootings, and other violent disasters have the potential to increase infectious disease through exposed wounds, bioweapons, forced migration, and the conglomeration of peoples from across the globe (49–53). Additionally, human activities such as fracking—fracturing of bedrock for oil production—can increase the incidence of natural disasters such as earthquakes, which may in turn increase incidence of earthquake-mediated infection (54). Other human-created disasters such as wastewater treatment mismanagement could have significant effects on microbes and their interactions with humans. Outbreaks of the gastrointestinal parasite *Cryptosporidium* have been associated with failures of wastewater treatment plants, which contaminated the water supply (55).

Other human-made disasters, particularly those that result from chronic and persistent human-made hazards, possess unusual and unnatural conditions that result in a unique microbiology in which microbes gain the ability to grow in extreme conditions. Examples include the colonization of the damaged reactor at Chernobyl with melanotic and radiophilic fungi (56–58). Microbiologists have studied some of these sites, and analysis of the microbial flora in these sites has provided unique insights. The association of melanotic fungi with the damaged reactor at Chernobyl led to experiments showing that melanized fungi grew faster during exposure to high radiation in a process that was postulated to be radiation capture by melanin and conversion into biologically useful energy (59). Similar adaptation has occurred in highly acidic and heavy metal-contaminated mining wastewater, where microbes can adapt to become acidophilic and heavy-metal resistant (60, 61). Bioremediating microbes isolated from Superfund sites—areas recognized as heavily polluted and in need of remediation—within the United States are adapted to degrade toxic bisphenols and dioxins, and those found in areas of high arsenic can recycle arsenic and prevent it from causing environmental damage (62, 63). The presence of benzene, toluene, ethylbenzene, and xylene (BTEX) in the environment, such as what occurs following a gasoline or oil spill, is associated with enrichment of BTEX-degrading and dechlorinating microbial activity at polluted sites (64). Like the changes in environmental microbiome following wildfires, environmental contamination at the urban Newtown Creek Superfund site was associated with unique aerosolized microbial composition, different from nonpolluted control sites (65). Such examples provide new insights into microbial physiology that can in turn inform research into exobiology, the study of life on other planets, by expanding the types of extreme environments that can sustain microbial life.

Stress adaptations to human-caused conditions can potentially facilitate infections of human hosts. For example, the antioxidant response to ionizing radiation could allow a microbe to resist immune-mediated oxidative bursts. Additionally, the ability to survive acidic conditions could allow survival within the host phagolysosome and evasion of degradation. Lastly, development of a spore coat or thickened cell wall can in theory allow resistance to immune stresses such as antimicrobial peptides, antimicrobial drugs, and oxidative damage.

## SOCIAL VULNERABILITIES AND ENVIRONMENTAL AND MICROBIAL INJUSTICE

As disasters are on the rise due to climate change and anthropogenic factors, we must consider those populations at greatest risk of experiencing climate crises and the downstream microbial effects. Their vulnerability makes them major stakeholders in the field of disaster microbiology and suggests that they warrant special attention in research and remediation strategies.

Low-income countries and the Global South (66) are expected to experience the most severe effects of climate change in the near future, including extreme heat disasters and flooding of rapidly-growing coastal cities (67–72). In the United States, communities of color and those with low socioeconomic status are disproportionately exposed to high levels of pollution and environmental contamination, and coastal cities are at high risk for climate disasters (43, 72, 73). Socially and economically vulnerable populations are disproportionally exposed to climate change and pollution, and thus environmental disasters, leading to environmental injustice. Environmental injustice is defined as “the disproportionate exposure of communities of color and the poor to pollution, and its concomitant effects on health and environment” (73). In the context of disaster microbiology, environmental injustice is inextricably linked to microbial injustice, which is defined as “inequitable microbial exposure and risk experienced by disadvantaged communities” (3, 74, 75), particularly used in reference to harmful, destructive, or pathogenic microbes. The same disadvantaged communities that are at enhanced risk of climate catastrophe are those most likely to be negatively impacted by microbes following disaster. This inequity is exacerbated by health inequalities in minority and low-income communities, potentially compounded by an inadequate access to health care, high rates of comorbidities, and immunocompromised states (76, 77). Faced with microbial exposure during a disaster, having a compromised immune system increases the risk of microbial infection—including with fungi and viruses that predominantly target immunocompromised individuals. Consideration of the priorities and issues of communities facing environmental and microbial injustice is a necessary tenet in the foundation of disaster microbiology and in framing the subsequent research aims and goals.

Disasters can cause social vulnerabilities through displacement. For example, unhoused people may be forced to reside in local shelters. This ties in directly to the principle of disaster microbiology, as displacement can expose people to crowded and unsanitary emergency conditions, which renders them vulnerable to communicable respiratory diseases that could spread readily (11, 12, 78). Drought conditions in Mexico were associated with migration to towns, where refugees lived in crowded shelters, leading to outbreaks of typhoid fever (79). Disasters also have the potential to cause large-scale migration of people and animals seeking safety and assistance, which can result in the spread of microbes from one region to another or cause illness in migrants being exposed to new disease-causing microbes where they settle. Current theories regarding the spread of the bubonic plague in Europe in the 14th century suggest that drought disasters caused plague-infected rodents to flee Asia and travel toward Europe, where the plague-causing bacterium was introduced (80). Foreign response to disaster with humanitarian aid and foreign assistance can also carry risk associated with disease outbreak. Following the 2010 earthquake in Haiti, epidemiological and molecular evidence suggest that United Nations peacekeeping personnel from Nepal may have inadvertently spread cholera to the nation of Haiti (81–83).

## PRINCIPLES, ORGANIZATION, AND EMERGENCE OF A NEW DISCIPLINE

**FIG 1:**
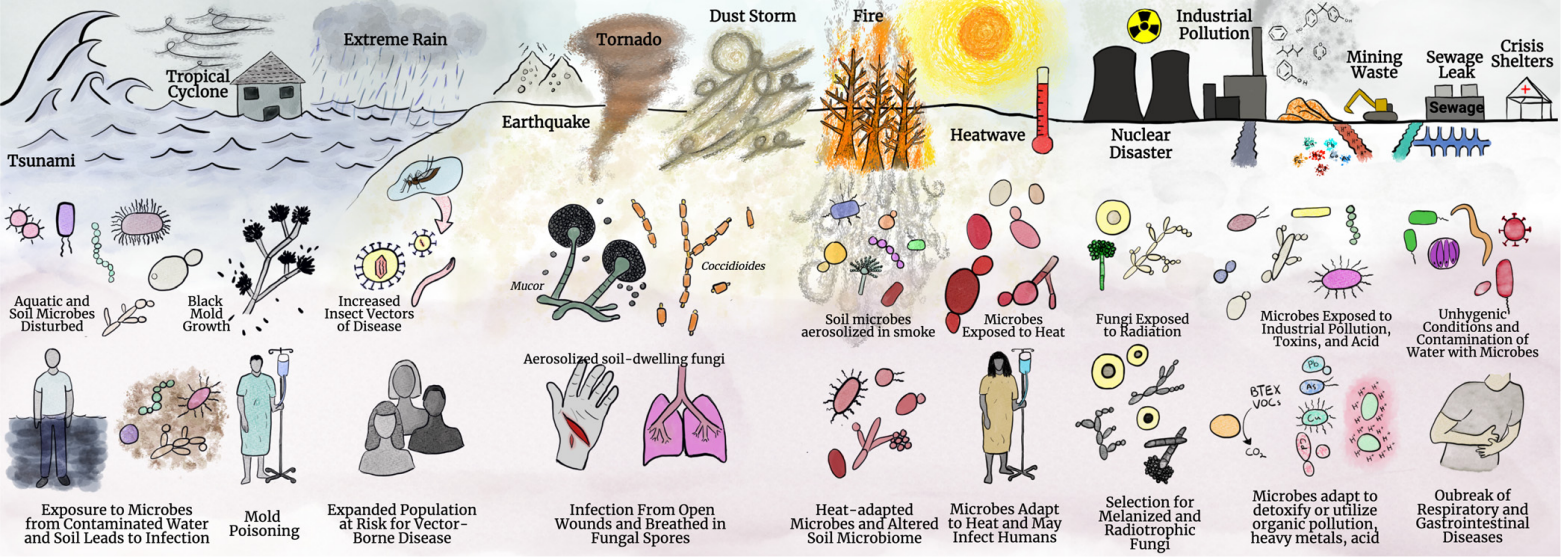
Overview of disaster microbiology. Disaster microbiology encompasses a diverse array of disasters, environmental changes, microbial adaptations, and secondary effects. Disaster microbiology includes microbial ramifications resulting from flooding disasters (tsunami, tropical cyclones, extreme rain), earthquakes, tornadoes, dust storms, wildfire, and droughts. Disaster microbiology also includes human-made disasters, which include chronic industrial pollution, nuclear disaster, sewage leaks, and humanitarian and refugee crises. These disasters are shown on the top row of the figure These disasters impact microbes and can disrupt microbial ecology and drive adaptations or alteration of the microbial population, as indicated in the graphics in the middle row. The alteration of microbial communities can then lead to disruption of human health, new ecological niches of microbes, and newly evolved biological processes that can be used in the future for bioremediation. The secondary effects are seen in the bottom row. Created with BioRender.com.

As is evident from the above discussion, disaster microbiology is an enormously diverse topic with the potential to emerge as a new field of study where natural and anthropogenic activities intersect to alter microbial communities in unanticipated ways with unanticipated consequences. The breadth of the field of disaster microbiology is summarized in Fig. 1. Given the human and natural antecedents for the disturbance leading to the disaster, disaster microbiology must be an interdisciplinary field that includes input from such disciplines as sociology, engineering, physics, chemistry, geology, climatology, and not least, microbiology. Further complexity follows the fact that each event is unique, but there are common themes that coalesce. We identify four contributing principles or criteria that we group together under the acronym DISEAASE to guide defining characteristics that serve as criteria for what falls under disaster microbiology.

**DIS**aster—a human or natural event must cause a disturbance or disaster or, “a sudden event… that causes great damage or loss of life” (1).

**E**nvironmental change—the disturbance alters the physical environment around it.

**A**daptation/**A**lteration—the changes in the environment promote adaptation and alterations for the microbial community (i.e., location/niche, stress, morphology).

**S**econdary **E**ffects—there is a consequence, or ramification, for the changes that occur in the microbial community. The microbe can gain virulence in a new host, permanently change its niche, colonize a structure or object, cause damage to infrastructure, provide insight into a new biological process, etc. Secondary effects include gaining new insights into microbial physiology.

Some examples of the DISEAASE guidelines for disaster microbiology for different disasters can be found in Table 1.

**TABLE 1:** Examples of the DISEAASE principles

Disaster (DIS)	Environmental change (E)	Microbial adaptation/alteration (AA)^*a*^	Secondary effect (SE)^*a*^	Ref
Rio Rinto mining runoff (3000 BC–Present)	Acidification of river and heavy metal contamination	Microbes are exposed to acidic environment	Microbes develop extreme pH tolerance	61
“El Año del Hambre” Mexico drought (1785)	Lack of water for crops; famine	Microbes carried by refugees in small unhygienic shelters	Typhus epidemic in rural refugees following famine	79
Portland Harbor Superfund site (1900s–Present)	Polychlorinated biphenyl and dioxin contamination	Microbes develop ways to degrade and use pollutants	New biological pathway to breakdown harmful pollutants; offers strategy for bioremediation	62
Great Alaskan Earthquake tsunami (1964)	Flood	Land colonization of Cryptococcus gattii	Outbreak of C. gattii in Pacific Northwest	27
California dust storm (1977)	Dust	Aerosolized *Coccidioides immitis*	Outbreak of coccidioidomycosis	32
Chernobyl meltdown (1986)	Radioactive contamination	Proliferation of melanized radiotrophic fungi	Colonization of reactor with radiotrophic fungi and discovery that melanin helps fungi acquire energy from radioactivity	56, 57
Milwaukee wastewater plant accident (1993)	Sewage contamination of water supply	Cryptosporidium enters water supply	Outbreak of cryptosporidiosis	55
California Northridge earthquake (1994)	Dust	Aerosolized *Coccidioides immitis*	Outbreak of coccidioidomycosis	33
Mozambique floods of 2000	Increased standing water	Increased proliferation of malaria vector mosquitoes	Increased incidence of malaria	28
2004 Indian Ocean tsunami	Flood; Abundance of donated resources	Extra supplies need to be stored in humid warehouse; growth of Aspergillus fumigatus on syringes	Syringes used for epidurals, results in outbreak of aspergillosis	84
Hurricane Katrina (2005)	Flood	Ideal environment for mold to form	Increase in “respiratory symptoms”	15
	Displacement of people	Microbes in a crowded evacuee shelter	Spread and outbreak of norovirus	85
2011 Japan Tsunami	Flood	*Legionella* spp. and A. fumigatus from soil found in water droplets	Legionellosis and aspergillosis outbreak	21, 22
Joplin tornado (2011)	Dust and wounds	Aerosolized *Apophysomyces trapeziformis*	Outbreak of mucormycosis	7
Global heat waves	Extreme heat	Heat-adapted Candida auris	Emergence of C. auris as a pathogen	9

a Some of the microbial adaptations and alterations and secondary effects listed in this table are proposed or hypothesized in the references provided, and additional research is needed for establishing causal relationships.

## CONCLUSIONS AND FUTURE DIRECTIONS

Disasters have long been studied as inciting incidents for outbreaks of infections and noncommunicable diseases. The disaster literature comes from many fields including epidemiology, medicine, microbiology, environmental health, and ecology. As other sectors of our society develop fields related to studying and mitigating disaster, the establishment of the disaster microbiology field could help fill a gap in research. Coalescing the work done in these separate fields into disaster microbiology is timely and will strengthen the organized efforts to study the disaster-mediated spread of microbes and infectious disease. Developing disaster microbiology symposia or tracks in scientific meetings could promote the emergence of this field.

One major component of disaster microbiology is how disasters physically change the location of microbes and the nature of their interactions with people. Disaster microbiology also delves into the evolution of microbes in response to environmental and disaster stress, and how that could have implications on health. Further work is needed to study the evolution of environmental microbes under natural and human-made disaster stress, particularly in vulnerable communities and regions. It will be vital to characterize how they have adapted to extreme heat and other urban conditions, such as through heat-stress mechanisms, melanin production, heavy metal detoxification, or radiation shielding. This can inform our understanding of (i) how disasters change the distribution and proliferation of microbes and their interaction with humans, (ii) how microbes are adapting to the stress and natural disasters as they occur, and (iii) how stress adaptation can drive a microbe’s ability to survive within human hosts and emerge as possible human pathogens.
